# Cooling the burn: EMLA alone vs. paravertebral block plus EMLA for improving tolerability of 8% capsaicin patch application in thoracic postherpetic neuralgia—a retrospective cohort study

**DOI:** 10.3389/fnagi.2026.1762229

**Published:** 2026-02-16

**Authors:** Hassan A. Moria

**Affiliations:** Department of Surgery, Faculty of Medicine, University of Tabuk, Tabuk, Saudi Arabia

**Keywords:** capsaicin, lidocaine, nerve block, pain management, postherpetic neuralgia, prilocaine, quality of life

## Abstract

**Background:**

The 8% capsaicin patch (Qutenza®) is an effective localized treatment for postherpetic neuralgia (PHN), yet intense application-related burning pain remains a major barrier to its broader use. Optimizing procedural tolerability is particularly important in elderly patients who are vulnerable to systemic analgesic side effects. Thoracic paravertebral block (TPVB) provides segmental analgesia and may attenuate procedural pain during capsaicin patch application.

**Objective:**

To compare procedural tolerability and short-term analgesic outcomes of topical EMLA alone versus EMLA combined with TPVB before 8% capsaicin patch application in patients with thoracic PHN.

**Methods:**

This retrospective cohort study included adults with thoracic PHN treated with 8% capsaicin patches at Prince Fahd bin Sultan Hospital, Tabuk, Saudi Arabia, between January 2022 and February 2025. Patients received either EMLA alone (*n =* 9) or EMLA plus ultrasound-guided TPVB (*n =* 8). The primary outcome was procedural tolerability, assessed using peak intraprocedural Numeric Pain Rating Scale (NPRS), area under the curve of NPRS over 60 min (AUC-NPRS), and need for intravenous (IV) tramadol. Secondary outcomes included NPRS at 8, 24, and 48 h; change in Pittsburgh Sleep Quality Index (PSQI); oral tramadol use over 48 h; and patient-reported acceptability.

**Results:**

Baseline characteristics were comparable between groups. Patients who received TPVB demonstrated markedly superior procedural tolerability compared with those pretreated with EMLA alone. Intraprocedural pain was substantially reduced, with a markedly lower cumulative pain burden and no requirement for IV rescue in the TPVB group, whereas all EMLA patients required opioid rescue and reported intense burning pain during application. Post-procedural pain scores remained consistently lower in the TPVB group, accompanied by significant improvements in sleep quality and reduced reliance on oral analgesics during the following 48 h. Treatment acceptability was also substantially higher with TPVB, indicating a considerably more comfortable peri-procedural experience.

**Conclusion:**

TPVB combined with EMLA yielded profound improvements in procedural tolerability, early pain control, sleep quality, and patient acceptability during 8% capsaicin patch therapy for thoracic PHN, while markedly reducing opioid rescue needs. These findings support TPVB-assisted capsaicin therapy as a promising multimodal strategy deserving evaluation in prospective controlled trials.

## Introduction

Postherpetic neuralgia (PHN) is a chronic neuropathic pain syndrome that follows herpes zoster and is particularly prevalent in older adults. It is associated with substantial sleep disturbance, mood impairment, functional limitation, and reduced quality of life (Jung and Park, 2016; Falcon, 2018; Adriaansen et al., 2024). The incidence of herpes zoster increases significantly with age, with approximately 50–60% of cases (95% CI 44–65%) of individuals who live to 85 years experiencing at least one episode, and PHN developing in 10–30% of these cases (Kim, 2024; Johnson and Rice, 2014). The burden of PHN extends beyond pain intensity to encompass profound impacts on sleep quality, physical functioning, and psychological well-being, creating a substantial public health challenge in aging populations (Schmader et al., 2007; Coplan et al., 2004).

Conventional pharmacologic options, including tricyclic antidepressants, gabapentinoids (gabapentin and pregabalin), and serotonin-noradrenaline reuptake inhibitors, are often limited by modest efficacy and adverse effects in elderly and comorbid populations (Finnerup et al., 2015; Dworkin et al., 2013; Rullán et al., 2017). Systemic medications carry risks of sedation, dizziness, cognitive impairment, and falls, particularly concerning in older adults with multiple comorbidities (Makris et al., 2014; American Geriatrics Society Panel on Pharmacological Management of Persistent Pain in Older Persons, 2009). Moreover, many patients experience inadequate pain relief despite optimized oral regimens, and treatment discontinuation rates remain high due to poor tolerability (Attal et al., 2010; Dworkin et al., 2010).

The 8% capsaicin patch (Qutenza®) provides a targeted topical alternative by inducing reversible defunctionalization of cutaneous nociceptors through prolonged activation of the transient receptor potential vanilloid 1 (TRPV1) channel (Burness and McCormack, 2016; Anand and Bley, 2011; Hall et al., 2020). This mechanism results in localized analgesia without systemic drug exposure, making it particularly attractive for elderly patients and those with contraindications to systemic agents (Blair, 2018; Hoy, 2020). Multiple randomized controlled trials and systematic reviews have demonstrated the efficacy of the 8% capsaicin patch in reducing PHN-related pain, with benefits persisting for up to 12 weeks following a single 60-min application (Backonja et al., 2008; Irving et al., 2011; Katz et al., 2015; Yong et al., 2017). Network meta-analyses comparing topical treatments for PHN have consistently ranked high-concentration capsaicin among the most effective options, with favorable risk–benefit profiles compared to systemic therapies (Liu et al., 2020; Guo et al., 2025).

Despite its proven efficacy, the clinical adoption of the 8% capsaicin patch has been limited by intense application-related burning pain, which can be intolerable for many patients (Laklouk and Baranidharan, 2016; Mou et al., 2013). During the application period, patients commonly experience severe procedural discomfort requiring rescue analgesia, and this barrier to tolerability has been identified as a major obstacle to broader implementation in clinical practice (Webster et al., 2010; Giaccari et al., 2021). Current guidelines recommend pre-treatment with topical local anesthetics, most commonly EMLA cream (eutectic mixture of lidocaine 2.5% and prilocaine 2.5%), to mitigate application pain (Derry et al., 2017; European Medicines Agency, 2020). However, clinical experience suggests that EMLA alone provides insufficient analgesia for many patients, particularly when treating large or highly sensitized areas (Haanpää et al., 2016; McCleane, 2000).

Regional anesthetic techniques, including paravertebral nerve blocks, have been widely used for perioperative analgesia and chronic pain management, providing segmental sensory blockade with minimal systemic effects (Richardson and Lönnqvist, 1998; Naja and Lönnqvist, 2001). Thoracic paravertebral block (TPVB) involves injection of local anesthetic into the paravertebral space, producing ipsilateral somatic and sympathetic nerve blockade across multiple dermatomes (Karmakar, 2001). This technique has demonstrated excellent safety and efficacy for managing acute and chronic thoracic pain conditions and may offer a novel approach to improving the tolerability of capsaicin patch application (Coveney et al., 1998; Lin et al., 2019).

To date, no published studies have systematically evaluated the role of paravertebral blockade in facilitating capsaicin patch application for PHN. Given the anatomical distribution of thoracic PHN and the segmental nature of paravertebral analgesia, we hypothesized that combining TPVB with standard EMLA pre-treatment would substantially improve procedural tolerability and early analgesic outcomes compared to EMLA alone. The present retrospective cohort study was undertaken to compare procedural pain intensity, rescue analgesic requirements, early pain relief, sleep quality improvement, and patient acceptability between these two approaches in patients with refractory thoracic PHN undergoing 8% capsaicin patch treatment.

## Methods

### Study design and setting

This retrospective observational cohort study was conducted at the Pain Clinic of Prince Fahd bin Sultan Hospital, Tabuk, Saudi Arabia. Medical records of all patients with thoracic postherpetic neuralgia (PHN) who received treatment with the 8% capsaicin patch between January 2022 and February 2025 were reviewed.

As treatment allocation was non-randomized and based on routine clinical judgment and patient preference, the study carries an inherent risk of selection bias.

### Patient selection

Medical records were reviewed to identify all adult patients (≥18 years) who received 8% capsaicin patch treatment for thoracic PHN during the study period. PHN was defined as persistent pain for ≥3 months following resolution of the herpes zoster rash in a dermatomal distribution. Inclusion criteria were: (1) confirmed diagnosis of thoracic PHN; (2) baseline Numeric Pain Rating Scale (NPRS) score ≥6/10 despite conventional pharmacotherapy more than 6 months; (3) complete documentation of pre-treatment anesthetic approach (EMLA alone or EMLA+TPVB); and (4) availability of procedural and 48-h follow-up data.

Exclusion criteria included: (1) allergy to local anesthetics or capsaicin; (2) active infection in the treatment area; (3) coagulopathy or anticoagulation therapy contraindicated for TPVB; (4) cognitive impairment preventing reliable pain assessment; and (5) PHN involving dermatomes other than thoracic.

### Treatment protocols

#### EMLA alone group

Patients in this group received standard pre-treatment with EMLA cream (lidocaine 2.5% + prilocaine 2.5%) applied as a thick layer over the entire treatment area and covered with an occlusive dressing for 60 min prior to capsaicin patch application. Following EMLA removal and cleansing, the 8% capsaicin patch (Qutenza®, Grünenthal) was applied to the affected area for 60 min under continuous monitoring.

#### EMLA + thoracic paravertebral block group

Patients in this group received ultrasound-guided TPVB in addition to EMLA pre-treatment. The TPVB was performed 10 min before EMLA application. With the patient in the sitting position, the thoracic paravertebral space was identified using a low-frequency convex probe (2–5 MHz) positioned 2–3 cm lateral to the midline at the appropriate thoracic level (determined by the dermatomal distribution of PHN). Following sterile preparation and local infiltration with 1% lidocaine, a 22-gauge, 80-mm needle was advanced in-plane under real-time ultrasound guidance until the tip was positioned in the paravertebral space, confirmed by visualization of the pleura displacement. A total of 7–10 mL of 0.25% bupivacaine with 40 mg triamcinolone was injected incrementally after negative aspiration.

Following TPVB, EMLA cream was applied as described above, and the capsaicin patch was subsequently applied for 60 min.

### Outcome measures

*Primary outcome: procedural tolerability*

Procedural tolerability was assessed using a composite of three measures:

1. Peak intraprocedural NPRS: The highest pain score recorded during the 60-min capsaicin application period. NPRS was assessed at 0, 10, 20, 30, 40, 50, and 60 min.
2. Area under the curve of NPRS (AUC-NPRS): Calculated using the trapezoidal rule to quantify cumulative pain exposure during the procedure.
3. Need for intravenous rescue analgesia: Documented use of IV tramadol (50–100 mg) for intolerable procedural pain.

*Secondary outcomes*

1. Post-procedural pain intensity: NPRS scores at 8, 24, and 48 h after patch removal.
2. Sleep quality: Change in Pittsburgh Sleep Quality Index (PSQI) from baseline to 48 h post-procedure. The PSQI is a validated 19-item questionnaire with scores ranging from 0 to 21, with higher scores indicating worse sleep quality.
3. Oral rescue analgesic use: Documented use of oral tramadol (50 mg as needed) during the 48-h post-procedural period.
4. Patient acceptability: Assessed at 48 h using a 5-point Likert scale (1 = completely unacceptable, 5 = completely acceptable).

### Data collection

Data were extracted from electronic medical records by two independent reviewers using a standardized data collection form. Variables collected included: demographics (age, sex), PHN characteristics (duration, dermatomal distribution, baseline NPRS), comorbidities, concurrent medications, procedural details, all outcome measures, and adverse events.

### Statistical analysis

Continuous variables are presented as mean ± standard deviation (SD) and were compared between groups using independent-samples t-tests after confirming normal distribution with the Shapiro–Wilk test. Categorical variables are presented as frequencies and percentages and were compared using Fisher’s exact test. The area under the NPRS curve was calculated using the trapezoidal method. Effect sizes were calculated using Cohen’s d for continuous outcomes.

All statistical tests were two-tailed, and *p*-values <0.05 were considered statistically significant. Given the exploratory nature of this retrospective cohort study, no adjustment for multiple comparisons was applied. Statistical analyses were performed using SPSS version 26.0 (IBM Corp., Armonk, NY). Because multiple outcomes and repeated time-points were evaluated, no adjustment for multiplicity was applied. The findings should therefore be interpreted as exploratory and hypothesis-generating. Ninety-five percent confidence intervals (95% CI) are reported to aid clinical interpretation beyond reliance on *p*-values alone.

### Ethical considerations

The study was conducted in accordance with the Declaration of Helsinki. This study was reviewed and approved by the Research Ethics Committee at the University of Tabuk (Approval Number UT-633-394-2025). Participation was voluntary, and informed consent was obtained from all respondents. Confidentiality and anonymity were maintained throughout the study.

## Results

### Patient characteristics

A total of 17 patients met the inclusion criteria and were included in the analysis: 9 patients in the EMLA alone group and 8 patients in the EMLA+TPVB group. Baseline demographic and clinical characteristics were well balanced between groups (Table 1). Mean age was 66.9 ± 5.9 years in the EMLA group and 66.9 ± 5.4 years in the EMLA+TPVB group (*p =* 0.99). The duration of PHN was similar between groups (21.8 ± 7.7 vs. 22.4 ± 8.8 months, *p =* 0.89), as was baseline pain intensity (NPRS 8.19 ± 0.29 vs. 8.04 ± 0.31, *p =* 0.32). All patients had failed prior treatment with at least two classes of systemic analgesics, most commonly gabapentinoids and tricyclic antidepressants.

**Table 1:** Baseline demographic and clinical characteristics.

Characteristic	EMLA (*n =* 9) Mean ± SD or *n* (%)	EMLA + TPVB (*n =* 8) Mean ± SD or *n* (%)	*p*-value
Age (years)	66.9 ± 5.9	66.9 ± 5.4	0.99
Sex (Male/Female)	5/4	4/4	0.99
PHN duration (months)	21.8 ± 7.7	22.4 ± 8.8	0.89
Baseline NPRS (0–10)	8.19 ± 0.29	8.04 ± 0.31	0.32
Dermatomes affected (n)	2.33 ± 0.50	2.50 ± 0.53	0.51
Prior gabapentinoid use	9 (100%)	8 (100%)	1.00
Prior TCA use	7 (77.8%)	6 (75.0%)	0.99

NPRS, Numeric Pain Rating Scale; PHN, postherpetic neuralgia; TCA, tricyclic antidepressant; TPVB, thoracic paravertebral block.

### Procedural tolerability

**Figure 1:**
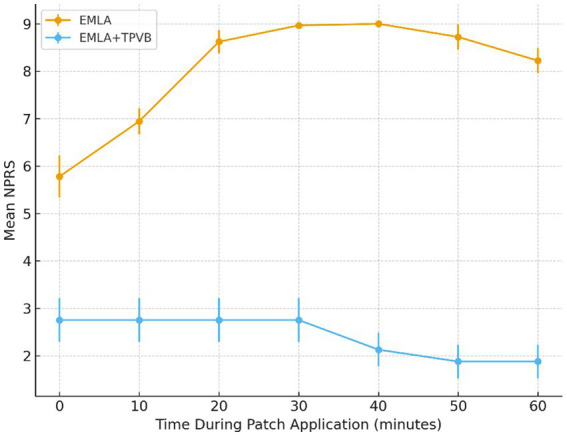
Intraprocedural pain trajectory (NPRS) during 8% capsaicin patch application. Mean NPRS scores at 0, 10, 20, 30, 40, 50, and 60 min in the EMLA group (*n =* 9) and the EMLA + TPVB group (*n =* 8). TPVB markedly blunted nociceptive activation, maintaining NPRS values between 2 and 3 throughout application. The EMLA group showed a steep rise in burning pain, peaking at 9/10 by 20–30 min. Error bars represent SD.

Peak intraprocedural NPRS (Figure 1) was 2.75 ± 0.46 in the EMLA+TPVB group compared to 9.0 ± 0.0 in the EMLA group (mean difference −6.25; 95% CI −6.63 to −5.87; *p =* 2.2 × 10^−9^).

**Figure 2:**
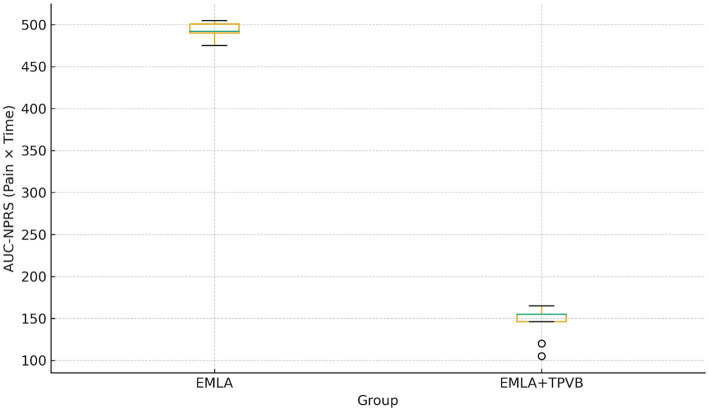
Area under the curve (AUC) for intraprocedural pain intensity. Boxplot comparison of cumulative pain burden over 60 min. AUC-NPRS was substantially lower in the TPVB group, indicating nearly a 70% reduction in pain exposure.

The cumulative pain burden, quantified by AUC-NPRS, was markedly lower with TPVB: 145.6 ± 21.1 versus 492.6 ± 10.8 in the EMLA group (*p =* 1.0 × 10^−12^; Cohen’s d = −21.1), representing an approximately 70% reduction in total pain exposure during the procedure (Figure 2). The between-group mean difference in AUC-NPRS was −347.0 (95% CI −365.5 to −328.5; *p =* 1.0 × 10^−12^).

The standardized effect size for procedural pain reduction was extremely large (Cohen’s d = 6.0; 95% CI 4.1–7.7).

**Figure 3:**
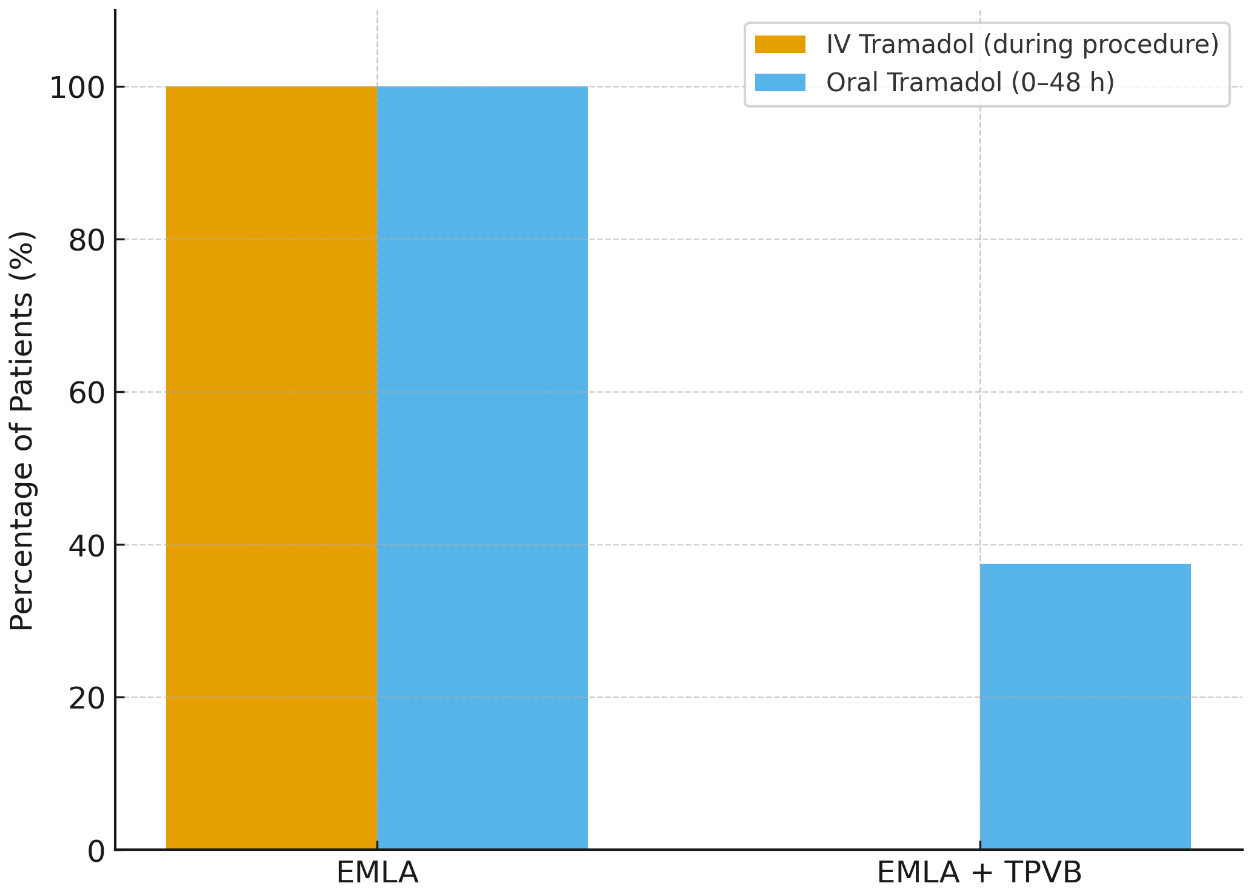
Rescue analgesic use (IV and oral tramadol). Proportion of patients requiring IV tramadol during application and oral tramadol within 48 h. All EMLA patients required IV rescue analgesia, whereas none in the TPVB group did. Post-procedural oral tramadol use was also significantly lower in the TPVB group.

All 9 patients (100%) in the EMLA alone group required IV tramadol rescue for intolerable procedural pain, administered at a median of 25 min (range 15–35 min) after patch application. In contrast, none of the 8 patients (0%) in the EMLA+TPVB group required IV rescue analgesia (*p =* 0.0003, Fisher’s exact test) (Figure 3).

### Post-procedural pain intensity

**Figure 4:**
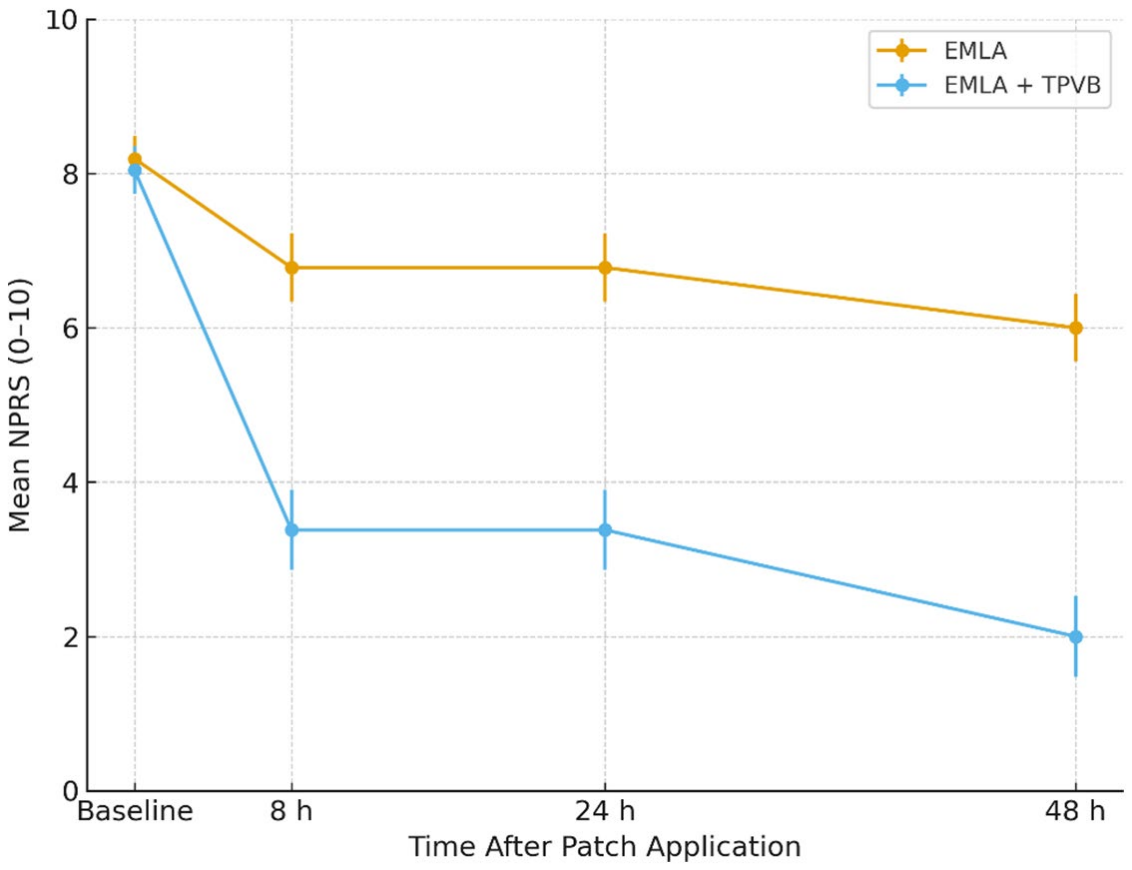
Post-procedural pain trajectory after capsaicin patch application. Mean NPRS at baseline, 8 h, 24 h, and 48 h in both groups. The TPVB group showed significantly lower pain across all timepoints, decreasing to NPRS 2.0 at 48 h compared with 6.0 in the EMLA group.

Pain relief was significantly greater in the EMLA+TPVB group at all post-procedural timepoints (Table 2; Figure 4). At 8 h post-procedure, mean NPRS was 3.38 ± 0.52 with TPVB versus 6.78 ± 0.44 with EMLA alone (*p =* 1.35 × 10^−11^). The between-group difference persisted at 24 h (2.38 ± 0.52 vs. 5.78 ± 0.44, *p =* 2.6 × 10^−11^) and 48 h (1.88 ± 0.35 vs. 5.67 ± 0.50, *p =* 1.35 × 10^−11^).

**Table 2:** Primary and secondary pain outcomes.

Outcome	EMLA (*n =* 9) Mean ± SD	EMLA + TPVB (*n =* 8) Mean ± SD	Mean difference (95% CI)	*p*-value
Peak intraprocedural NPRS	9.0 ± 0.0	2.75 ± 0.46	−6.25 (95% CI −6.63 to −5.87)	2.2 × 10^−9^
AUC-NPRS (0–60 min)	492.6 ± 10.8	145.6 ± 21.1	−347.0 (95% CI −365.5 to −328.5)	1.0 × 10^−12^
IV tramadol required (n, %)	9 (100%)	0 (0%)	—	0.0003
NPRS at 8 h	6.78 ± 0.44	3.38 ± 0.52	−3.40	1.35 × 10^−11^
NPRS at 24 h	5.78 ± 0.44	2.38 ± 0.52	−3.40	2.6 × 10^−11^
NPRS at 48 h	5.67 ± 0.50	1.88 ± 0.35	−3.79 (95% CI −4.24 to −3.34)	< 0.001
ΔNPRS (8 h – baseline)	−1.41 ± 0.53	−4.66 ± 0.58	—	1.35 × 10^−11^
ΔNPRS (24 h – baseline)	−2.41 ± 0.53	−5.66 ± 0.58	—	2.6 × 10^−11^
ΔNPRS (48 h – baseline)	−2.52 ± 0.44	−6.16 ± 0.39	—	1.35 × 10^−11^

Effect size note: Procedural pain reduction Cohen’s d = 6.0 (95% CI 4.1–7.7). AUC, area under the curve; NPRS, Numeric Pain Rating Scale; IV, intravenous; TPVB, thoracic paravertebral block.

The magnitude of pain reduction from baseline was also significantly greater with TPVB. At 48 h, the EMLA+TPVB group achieved a mean pain reduction of 6.16 ± 0.39 points on the NPRS compared to 2.52 ± 0.44 points in the EMLA group (*p =* 1.35 × 10^−11^). At 48 h, the between-group difference in NPRS was −3.79 (95% CI −4.24 to −3.34; *p* < 0.001).

### Sleep quality

Baseline PSQI scores were similar between groups (14.33 ± 1.00 vs. 14.0 ± 1.31, *p* > 0.05), indicating severely impaired sleep quality in both cohorts (Table 3). At 48 h post-procedure, PSQI improved markedly in the EMLA+TPVB group (9.0 ± 0.93) but remained essentially unchanged in the EMLA group (14.11 ± 0.78). The mean change in PSQI was −5.0 ± 0.53 points with TPVB versus −0.22 ± 0.44 points with EMLA alone, yielding a mean between-group difference of −4.78 points (95% CI −5.25 to −4.31; *p =* 1.7 × 10^−11^), representing a clinically meaningful improvement in sleep quality.

**Table 3:** Sleep, rescue oral tramadol use, and acceptability.

Outcome	EMLA (*n =* 9) Mean ± SD or *n* (%)	EMLA + TPVB (*n =* 8) Mean ± SD or *n* (%)	Effect estimate (95% CI)	*p*-value
PSQI baseline	14.33 ± 1.00	14.0 ± 1.31	—	0.72
PSQI at 48 h	14.11 ± 0.78	9.0 ± 0.93	—	< 0.001
ΔPSQI (48 h–baseline)	−0.22 ± 0.44	−5.0 ± 0.53	−4.78 (95% CI −5.25 to −4.31)	1.7 × 10^−11^
Any oral tramadol use (0–48 h)	9 (100%)	3 (37.5%)	Relative risk 0.41 (95% CI 0.18–0.94)	0.022
Acceptability (Likert 1–5)	1.44 ± 0.53	4.63 ± 0.52	Mean difference 3.19 (95% CI 2.69–3.69)	2.7 × 10^−9^

Effect size note: Acceptability Cohen’s d = 6.07. PSQI, Pittsburgh Sleep Quality Index; TPVB, thoracic paravertebral block.

### Oral rescue analgesic use

All 9 patients (100%) in the EMLA group required oral tramadol during the 48-h post-procedural period compared with 3 of 8 patients (37.5%) in the EMLA+TPVB group (*p =* 0.022, Fisher’s exact test) (Figure 3; Table 3). This corresponded to a relative risk of 0.41 (95% CI 0.18–0.94) for oral tramadol use with TPVB compared with EMLA alone, indicating a substantial opioid-sparing effect.

### Patient acceptability

**Figure 5:**
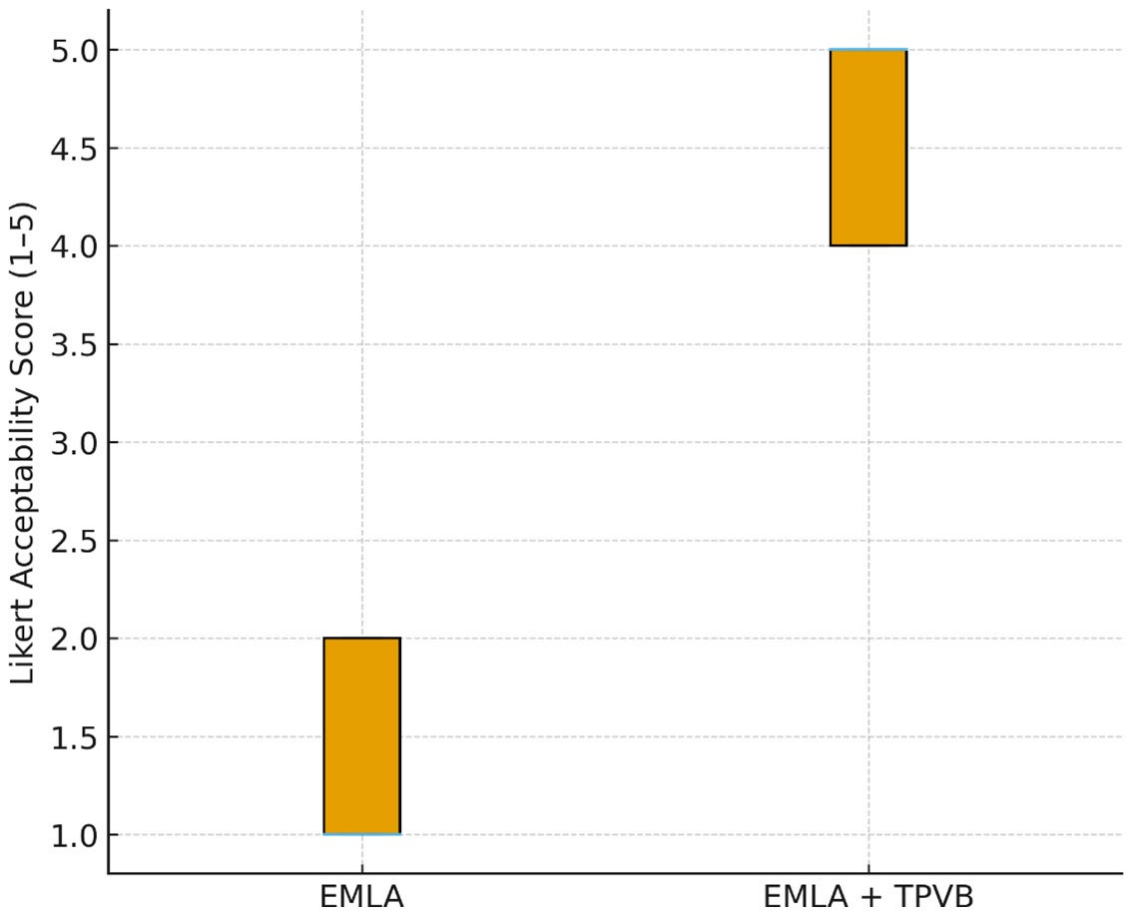
Patient acceptability of capsaicin patch application. Distribution of 5-point Likert acceptability scores. The TPVB group reported uniformly high tolerability, while the EMLA group reported poor procedural comfort.

Patient-reported acceptability of the procedure differed markedly between groups (Table 3; Figure 5). The mean acceptability score was 4.63 ± 0.52 in the EMLA+TPVB group compared to 1.44 ± 0.53 in the EMLA group. The mean difference in acceptability scores was 3.19 (95% CI 2.69–3.69; *p =* 2.7 × 10^−9^). The standardized effect size was extremely large (Cohen’s d = 6.07), indicating a profound improvement in perceived tolerability. All TPVB patients rated the procedure as acceptable or highly acceptable (scores 4–5), whereas all EMLA-only patients rated it as unacceptable or barely tolerable (scores 1–2).

### Safety

No serious adverse events were observed in either group. No pneumothorax, vascular puncture, or neurological complications related to TPVB were observed. Local application-site reactions (erythema, edema) occurred in both groups but were mild and self-limited.

## Discussion

This retrospective cohort study demonstrates that thoracic paravertebral block combined with EMLA cream dramatically improves the tolerability of 8% capsaicin patch application in patients with thoracic PHN compared to EMLA alone. The addition of TPVB resulted in a 70% reduction in cumulative procedural pain burden, eliminated the need for intravenous rescue analgesia, and was associated with superior early pain relief, improved sleep quality, reduced oral opioid consumption, and markedly higher patient acceptability.

The intense burning pain during capsaicin patch application represents a well-recognized barrier to the broader clinical use of this effective therapy (Laklouk and Baranidharan, 2016; Webster et al., 2010). Despite the proven efficacy of the 8% capsaicin patch in reducing PHN-related pain for up to 12 weeks (Backonja et al., 2008; Irving et al., 2011; Katz et al., 2015), patient and clinician reluctance related to application discomfort has limited its adoption in routine practice (Giaccari et al., 2021; Haanpää et al., 2016). Our findings confirm that standard EMLA pre-treatment, while universally recommended in clinical guidelines (Derry et al., 2017; European Medicines Agency, 2020), provides inadequate procedural analgesia for many patients—all nine patients in our EMLA-only group required IV opioid rescue, and all rated the procedure as unacceptable or barely tolerable.

The mechanism underlying capsaicin-induced pain involves intense activation of TRPV1 receptors on cutaneous nociceptors, triggering calcium influx, neuropeptide release, and robust firing of peripheral and central pain pathways (Burness and McCormack, 2016; Anand and Bley, 2011). EMLA provides superficial dermal anesthesia through sodium channel blockade but may not achieve sufficient depth of penetration or duration of action to fully suppress the nociceptive barrage generated by high-concentration capsaicin (McCleane, 2000). In contrast, paravertebral blockade interrupts nociceptive transmission at the level of the spinal nerve roots, providing profound segmental analgesia that encompasses both superficial and deep structures (Karmakar, 2001; Coveney et al., 1998).

Our results demonstrate the clinical superiority of this multimodal approach: TPVB patients maintained low pain scores (NPRS 2–3) throughout the entire 60-min application, whereas EMLA-only patients experienced rapid escalation to maximal pain intensity. This difference translated into a profound reduction in cumulative pain exposure (AUC-NPRS) and elimination of IV rescue requirements. From a patient-centered perspective, the acceptability ratings underscore the transformative impact of TPVB on the treatment experience.

Beyond procedural tolerability, the EMLA+TPVB group demonstrated significantly greater pain relief at all post-procedural timepoints through 48 h. At 48 h, TPVB patients achieved a mean 6.2-point reduction in NPRS compared to 2.5 points with EMLA alone. This enhanced early response may reflect several mechanisms. First, by minimizing procedural nociceptive input, TPVB may reduce central sensitization and wind-up phenomena that can amplify post-procedural pain (Woolf, 2011). Second, the residual analgesic effects of ropivacaine (duration 8–12 h) may have bridged the initial post-application period before capsaicin-induced nociceptor defunctionalization fully manifests (McClure, 1996). Third, improved procedural tolerability may have allowed more complete and uniform capsaicin delivery to the affected dermatomes, optimizing therapeutic effect.

The magnitude of pain reduction observed in the TPVB group is consistent with the response rates reported in randomized controlled trials of the 8% capsaicin patch (Irving et al., 2011; Yong et al., 2017; Liu et al., 2020). Network meta-analyses comparing topical agents for PHN have demonstrated that high-concentration capsaicin produces superior pain relief compared to lidocaine patches and comparable or superior outcomes to systemic gabapentinoids, with the advantage of localized delivery and absence of systemic side effects (Liu et al., 2020; Guo et al., 2025). Our findings suggest that optimizing procedural tolerability through regional anesthesia may enhance the early clinical benefit of capsaicin treatment.

Sleep disturbance is a cardinal feature of PHN, with the majority of patients reporting significant impairment in sleep quality that contributes substantially to overall disease burden (Schmader, 2007; Drolet et al., 2010). Chronic neuropathic pain disrupts sleep architecture through multiple mechanisms, including nocturnal pain exacerbations, anxiety, and central nervous system hyperarousal (Smith and Haythornthwaite, 2004). Conversely, poor sleep quality amplifies pain perception and impairs coping mechanisms, creating a bidirectional relationship between pain and sleep dysfunction (Finan et al., 2013).

In our cohort, baseline PSQI scores indicated severe sleep impairment in both groups. The dramatic improvement in PSQI observed in the TPVB group (mean reduction of 5.0 points at 48 h) represents a clinically meaningful change exceeding established minimal important difference thresholds (Buysse et al., 1989). This improvement likely reflects the combined effects of superior pain relief and reduced opioid consumption (discussed below). In contrast, the EMLA-only group showed minimal sleep improvement despite some pain reduction, potentially due to the traumatic procedural experience, higher residual pain levels, and greater opioid use.

The rapid improvement in sleep quality observed with effective PHN treatment has important implications for patient functioning and quality of life. Systematic reviews have consistently identified sleep disturbance as a major determinant of quality of life in PHN patients, often correlating more strongly with functional impairment than pain intensity alone (Oster et al., 2005; Pickering and Leplège, 2011). Interventions that provide rapid pain relief and sleep restoration may therefore offer particular value in this population.

A key advantage of the TPVB-assisted approach was the dramatic reduction in opioid requirements, both intraprocedurally and during the 48-h post-procedural period. No TPVB patient required IV tramadol during the procedure, compared to 100% of EMLA-only patients. Similarly, oral tramadol consumption was reduced by more than 60% in the TPVB group during the early recovery period.

The opioid-sparing potential of regional anesthesia techniques is well established in perioperative and chronic pain settings (Joshi et al., 2008; Baidya et al., 2014). Minimizing opioid exposure is particularly important in elderly PHN patients, who are at heightened risk for opioid-related adverse effects including sedation, cognitive impairment, falls, constipation, and respiratory depression (Pergolizzi et al., 2008; Gloth, 2011). Moreover, even short-term opioid use in opioid-naïve elderly patients carries risks of prolonged use and dependence (Shah et al., 2017).

The combination of TPVB with capsaicin patch treatment represents an attractive opioid-sparing multimodal analgesic strategy. By providing effective procedural and early post-procedural analgesia without systemic medications, this approach may facilitate access to capsaicin therapy for patients with contraindications to opioids or those at high risk for adverse effects (Manchikanti et al., 2017; Dowell et al., 2016). From a healthcare systems perspective, reducing opioid consumption aligns with broader public health initiatives to minimize opioid prescribing and combat the ongoing opioid crisis (Volkow and McLellan, 2016).

Our findings contribute to a growing body of literature supporting the use of regional anesthetic techniques to facilitate interventional pain procedures (Abdallah et al., 2014; Schnabel et al., 2010). Paravertebral blocks have been successfully employed to improve the tolerability of various painful procedures, including rib fracture management, chest wall interventions, and breast surgery (Karmakar et al., 2003; Davies et al., 2006). The present study extends this evidence base to the specific application of facilitating capsaicin patch treatment for PHN.

The thoracic paravertebral space is anatomically well-suited for managing thoracic neuropathic pain, as it allows for targeted blockade of multiple contiguous dermatomes with a single injection or a small number of injections (Karmakar, 2001; Lönnqvist et al., 1995). Ultrasound guidance has enhanced the safety and reliability of TPVB, allowing real-time visualization of anatomical structures and needle trajectory, thereby reducing the risk of complications such as pneumothorax or vascular puncture (Hara et al., 2009; Shibata and Nishiwaki, 2009). In our series, no serious complications related to TPVB were observed, consistent with the excellent safety profile reported in contemporary literature (Pace et al., 2016).

Systematic reviews of interventional treatments for PHN have identified several promising approaches, including nerve blocks, pulsed radiofrequency, and neuromodulation techniques (Lin et al., 2019; Isagulyan et al., 2023). However, most of these interventions are employed as standalone treatments for refractory PHN rather than as adjuncts to enhance the tolerability of other therapies. Our study introduces a novel paradigm: using regional anesthesia not as a primary treatment but as an enabling technique to facilitate delivery of a highly effective topical therapy that would otherwise be poorly tolerated.

Various strategies have been proposed to improve capsaicin patch tolerability, including cooling devices, opioid premedication, and anxiolytic agents (Webster et al., 2010; Haanpää et al., 2016). However, these approaches have shown limited efficacy or carry their own risks and side effects. Cooling may partially counteract capsaicin-induced TRPV1 activation but provides inconsistent relief and may interfere with patch adhesion (Minson et al., 2001). Oral or parenteral opioids can reduce procedural pain but introduce systemic side effects, particularly problematic in elderly patients (Pergolizzi et al., 2008; Gloth, 2011).

Topical anesthetics remain the standard of care, with EMLA being the most widely studied and recommended agent (Derry et al., 2017; European Medicines Agency, 2020). Some centers have explored higher-concentration or longer-duration topical anesthetic applications, but these modifications carry increased risks of systemic local anesthetic toxicity, particularly when applied to large surface areas. Alternative topical agents, such as 4% lidocaine patches or compounded high-concentration lidocaine creams, have been investigated but have not demonstrated clear superiority over EMLA for capsaicin pre-treatment (Wang et al., 2023).

Regional anesthesia offers distinct advantages over these alternatives: profound analgesia without systemic drug exposure, extended duration of action, and established safety when performed with ultrasound guidance (Hara et al., 2009; Shibata and Nishiwaki, 2009; Pace et al., 2016). The addition of TPVB to standard EMLA pre-treatment represents a synergistic multimodal approach, combining superficial dermal anesthesia with deep segmental blockade to comprehensively suppress nociceptive transmission during capsaicin application.

Our findings suggest that TPVB-assisted capsaicin patch application may represent an optimal treatment strategy for patients with thoracic PHN, particularly those who have failed conservative therapies and require an effective, well-tolerated intervention. This approach may be especially valuable for elderly patients, those with multiple comorbidities, individuals with contraindications to systemic analgesics, and patients who have previously declined or discontinued capsaicin treatment due to tolerability concerns.

Implementation of TPVB-assisted capsaicin therapy requires access to practitioners skilled in ultrasound-guided regional anesthesia, appropriate monitoring equipment, and adequate time for the procedure (TPVB followed by EMLA application and capsaicin patch placement). In specialized pain management centers, these resources are typically available, and the incremental time and cost associated with TPVB may be offset by improved patient outcomes, reduced rescue medication use, and higher treatment acceptance and adherence.

From a healthcare economics perspective, the cost-effectiveness of TPVB-assisted capsaicin therapy merits formal evaluation. While the addition of TPVB increases procedural complexity and resource utilization, the potential benefits include reduced opioid consumption, fewer complications related to systemic medications, improved patient satisfaction, and potentially enhanced long-term efficacy of capsaicin treatment. Pharmacoeconomic analyses have demonstrated the cost-effectiveness of the 8% capsaicin patch compared to conventional systemic therapies for PHN (Armstrong et al., 2011; Boyle et al., 2012); extending such analyses to include TPVB-assisted application would be valuable (Chen et al., 2020; Liu et al., 2021; Pei et al., 2019; Dhikav et al., 2016).

### Limitations

Several limitations of this study warrant acknowledgment. First, as a retrospective non-randomized cohort study, the possibility of selection bias cannot be excluded. Although baseline characteristics were comparable, patients who received TPVB may have differed in unmeasured characteristics from those receiving EMLA alone. Second, because multiple endpoints and time-points were analyzed, no formal adjustment for multiple comparisons was performed, which may increase the risk of type I error; accordingly, results should be regarded as exploratory. Third, outcome assessments were limited to 48 h, preventing evaluation of medium- and long-term analgesic efficacy after capsaicin therapy, future studies should examine whether improved procedural tolerability with TPVB translates into enhanced long-term efficacy.

Finally, the sample size was small (*n =* 17), reflecting the single-center nature of the study and the relatively limited use of capsaicin patches in routine clinical practice. While the magnitude of between-group differences was large and highly statistically significant, the small sample limits the generalizability of findings and precludes robust subgroup analyses. Larger multicenter studies are needed to confirm these results across diverse patient populations and practice settings.

### Future directions

These findings highlight the need for prospective randomized controlled trials to confirm the efficacy, safety, and cost-effectiveness of TPVB-assisted capsaicin patch application for thoracic PHN. Future studies should incorporate longer-term follow-up to evaluate sustained analgesic benefit, quality of life, treatment adherence, and economic outcomes, as well as compare TPVB-assisted therapy with other interventional options for refractory PHN. Exploration of alternative regional anesthetic techniques for cervical, lumbar, or trigeminal PHN is warranted. Mechanistic studies using sensory testing or neuroimaging may clarify how regional anesthesia modulates capsaicin-induced nociceptor defunctionalization. Finally, implementation research addressing training, workflow, and patient preferences will be essential for integrating TPVB-assisted capsaicin therapy into routine practice.

## Conclusion

This retrospective cohort study demonstrates that thoracic paravertebral block combined with EMLA cream dramatically improves the tolerability of 8% capsaicin patch application in patients with thoracic PHN compared to EMLA alone. The addition of TPVB resulted in profound reductions in procedural pain, elimination of intravenous opioid rescue requirements, superior early pain relief, meaningful improvements in sleep quality, reduced oral opioid consumption, and markedly higher patient acceptability. TPVB-assisted capsaicin therapy represents a promising opioid-sparing multimodal strategy for patients with refractory thoracic PHN. These findings support the need for prospective randomized controlled trials to confirm efficacy, assess long-term outcomes, and evaluate cost-effectiveness of this approach. If validated in larger studies, TPVB-assisted capsaicin patch application may become a preferred treatment option for patients with thoracic PHN, particularly elderly individuals and those with contraindications to systemic analgesics.

## Data Availability

The data supporting the findings of this study are included within the article, further inquiries should be directed to the corresponding author/s.
